# A vasculature niche orchestrates stromal cell phenotype through PDGF signaling: Importance in human fibrotic disease

**DOI:** 10.1073/pnas.2120336119

**Published:** 2022-03-23

**Authors:** Thomas B. Layton, Lynn Williams, Nan Yang, Mingjun Zhang, Carl Lee, Marc Feldmann, Glenda Trujillo, Dominic Furniss, Jagdeep Nanchahal

**Affiliations:** ^a^The Kennedy Institute of Rheumatology, Nuffield Department of Orthopaedics, Rheumatology and Musculoskeletal Sciences, University of Oxford, Oxford OX3 7FY, United Kingdom;; ^b^Biotherapeutics, Bristol-Myers Squibb, San Diego, CA 92121;; ^c^Fibrosis Biology Drug Discovery, Bristol-Myers Squibb, Lawrenceville, NJ 08648;; ^d^The Botnar Research Centre, Nuffield Department of Orthopaedics, Rheumatology and Musculoskeletal Sciences, University of Oxford, Oxford OX3 7LD, United Kingdom

**Keywords:** fibrosis, vasculature, single-cell sequencing, PDGF, inflammation

## Abstract

Fibrosis is characterized by excessive matrix protein accumulation and contributes to significant morbidity and mortality in the Western world. The relative lack of effective antifibrotic therapeutics for the majority of these conditions reflects the difficulty in identifying targets for human fibrosis. Animal models fail to recapitulate all of the facets of human disease, and the limited clinical samples from patients with fibrosis of visceral organs are usually of late-stage disease [J. Nanchahal, B. Hinz, *Proc. Natl. Acad. Sci. U.S.A.* 113, 7291–7293 (2016)]. Here, we use Dupuytren’s disease (DD), a localized fibrotic condition of the hand, as a model to profile the vasculature niche of human fibrosis at single-cell resolution. Our spatially resolved molecular taxonomy of fibrotic blood vessels identifies distinct endothelial and pericyte populations and demonstrates a complex topological organization in the fibrotic microenvironment. In developing fibrosis, we show that the endothelium acts to promote immune regulatory fibroblast phenotype through platelet-derived growth factor (PDGF) signaling, thereby sustaining an immune cell–enriched perivascular niche. Moreover, we highlight pericytes as “housing” a putative myofibroblast precursor in DD. Overall, our results elucidate a tightly coupled vasculature niche in fibrosis that instructs the differentiation of functionally distinct stromal cells. These findings provide an important translational resource and highlight the therapeutic potential of targeting blood vessel signaling in human fibrosis.

## Significance

Tissue fibrotic diseases, for example of the liver and lung, represent a huge unmet medical need. In this study, using single-cell RNA sequencing, cytometry by time of flight (CyTOF), tissue imaging, and functional assays, we identify a complex vascular niche in Dupuytren’s disease (DD), a common localized fibrotic condition of the palm, where early-disease-stage tissue can be accessed readily. We uncover a population of myofibroblast precursors within the pericyte compartment and demonstrate that the endothelium instructs the differentiation of functionally distinct stromal cells, thereby orchestrating discrete microenvironments in the fibrotic milieu. Together, these findings provide a basis for the concept of targeting blood vessel signaling to control the progression of human fibrosis.

Tissue fibrosis is characterized by excessive extracellular matrix protein accumulation and, as it can affect many organs, contributes to significant morbidity and mortality in the Western world (1–3). Collagen-producing stromal cells are central to the pathogenesis of fibrosis (4, 5), and their activation drives a coordinated pathogenic sequence that ultimately compromises organ function (1, 2). Despite intensive research efforts, effective antifibrotic therapeutics are currently missing (3). One challenge in studying fibrosis is the limited availability of well-characterized early-stage patient samples (3, 6), and reliance on murine models of fibrosis has failed to identify meaningful targets for human disease. A comprehensive understanding of the human fibrotic microenvironment during the early stages of the disease would greatly facilitate identification of targets more likely to successfully translate to the clinic (7–11). In visceral fibrosis, studies are restricted to small biopsy samples with inherent sampling bias, supporting the value of detailed profiling of localized human fibrosis for the precise classification of the cellular landscape as fibrosis develops.

Localized fibrotic diseases provide a valuable source of early-stage human tissue. Dupuytren’s disease (DD) is a common and progressive fibroproliferative disorder of the palmar fascia of the hand and in Western populations affects 29% of those 75 y and older (12). The initial clinical presentation is the appearance of firm nodules in the palm, which expand into fibrous collagenous cords that extend into the digits. Dupuytren’s nodules, which represent the early stage of the disease, are a highly cellular fibrotic structure (13, 14) and represent a model to study developing fibrosis in humans (15).

In this study, using clinical samples from DD patients, we resolve the distinct anatomical and transcriptomic profile of the vascular niche in human fibrosis. Our single-cell atlas elucidates distinct pericytes and endothelial subsets. Spatially mapping the vascular niche shows how this compartment acts to sustain fibroblast identity and maintain an immune regulatory fibroblast microenvironment, as well as identifying a role for platelet-derived growth factor (PDGF) signaling in fibrosis. In addition, we show that pericytes are a putative myofibroblast precursor, including an intermediate cell state. These findings provide translational opportunities for targeting DD and potentially other fibrotic diseases such as interstitial pulmonary fibrosis (IPF).

## Results

### Single-Cell Atlas of the Vascular Niche in Human Fibrosis.

**Fig. 1.**
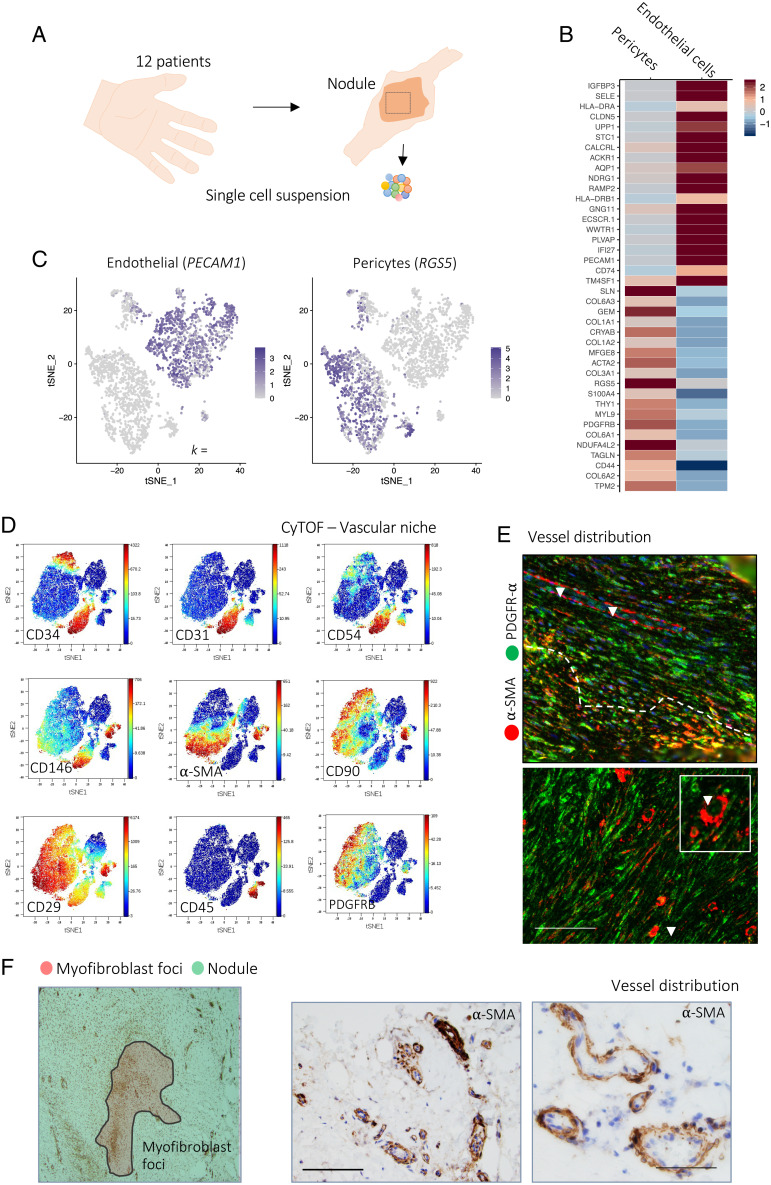
Single-cell atlas of the vascular niche in human fibrosis. (*A*) Schematic illustrating the experimental protocol for single-cell profiling of Dupuytren’s nodule. (*B*) Heatmap of scRNA-seq showing a *z* score–normalized mean expression of cell-type marker genes (*n* = 12 DD patients). (*C*) tSNE projections of scRNA-seq for 7,223 cells showing pericytes (*RGS5* expression) and endothelial cells (*PECAM1* expression) in Dupuytren’s nodules. (*D*) tSNE projections of CyTOF analysis from a representative Dupuytren’s nodule, colored by normalized protein expression of cell-type markers (*n* = 6 DD patients). (*E*) Confocal images of immunofluorescence showing expression of α-SMA (pericytes) and PDGFR-α (fibroblasts) in DD nodules (*n* = 6 DD patients). (Scale bar, 20 µm.) Arrrowheads indicate blood vessels. (*F*) Microscopy images of immunohistochemistry showing expression of α-SMA (pericytes) in DD nodules (*n* = 6 DD patients). (Scale bars, 60 µm.).

To investigate vasculature heterogeneity during human fibrosis, we first performed paired single-cell RNA sequencing (scRNA-seq) and mass cytometry (cytometry by time of flight [CyTOF]) on vascular cells isolated directly from Dupuytren’s nodules, generating a dataset yielding high-quality profiles of over 100,000 cells from 18 patients (12 scRNA-seq and 6 CyTOF) (Fig. 1 *A*–*D* and *SI Appendix*, Fig. S1). Initially, we employed graph-based clustering in DD nodules to identify discrete populations of endothelial cells and pericytes. Pericytes and endothelial cells separated into distinct clusters, with discrete transcriptomic signatures (Fig. 1*B*). We then undertook detailed characterization of vascular cells, defining key protein and messenger RNA (mRNA) markers of endothelial cells (CD31/PECAM1, CD34, ICAM1/CD54) and pericytes (CD146, α-SMA, CD29/ITGβ1) (Fig. 1*C*) using CyTOF and scRNA-seq. Next, using flow cytometry of freshly isolated nodular cells, we validated discrete populations of CD146^+^ pericytes and CD31^+^ endothelial cells in DD nodules and, correlating with the scRNA-seq data, demonstrated each represented ∼5 to 10% of the total stromal cell pool (*SI Appendix*, Fig. S2).

We then sought to spatially map the vascular niche in the DD nodules (*SI Appendix*, Figs. S3 and S4). We noted DD nodules contained foci consisting of densely packed α-SMA^+^ myofibroblasts, analogous to those seen in idiopathic pulmonary fibrosis (*SI Appendix*, Fig. S4). We used immunohistochemical staining for α-SMA to characterize pericyte-lined vessels in whole DD nodule tissue sections (Fig. 1 *E* and *F*). Surprisingly, this demonstrated vessels were predominantly located outside the myofibroblast foci. Vessels were instead distributed within areas of α-SMA^−^ fibroblasts and, although heterogeneous, most frequently had a small and convoluted morphology (*SI Appendix*, Fig. S2 *D* and *E*). The distribution of vessels outside the myofibroblast foci suggested a closer association with fibroblasts, and we sought to examine this further. Using multiplex immunofluorescence microscopy, we confirmed that the vascular niche was indeed significantly closer to fibroblasts (PDGFRA^+^α-SMA^−^) than the myofibroblast (PDGFRA^−^α-SMA^+^) foci (*SI Appendix*, Fig. S2*E*). Together, these results established a vascular niche in developing human fibrosis that resides in close proximity to α-SMA^−^PDGFRA^+^ fibroblasts.

### Pericytes House a Putative Myofibroblast Precursor.

**Fig. 2.**
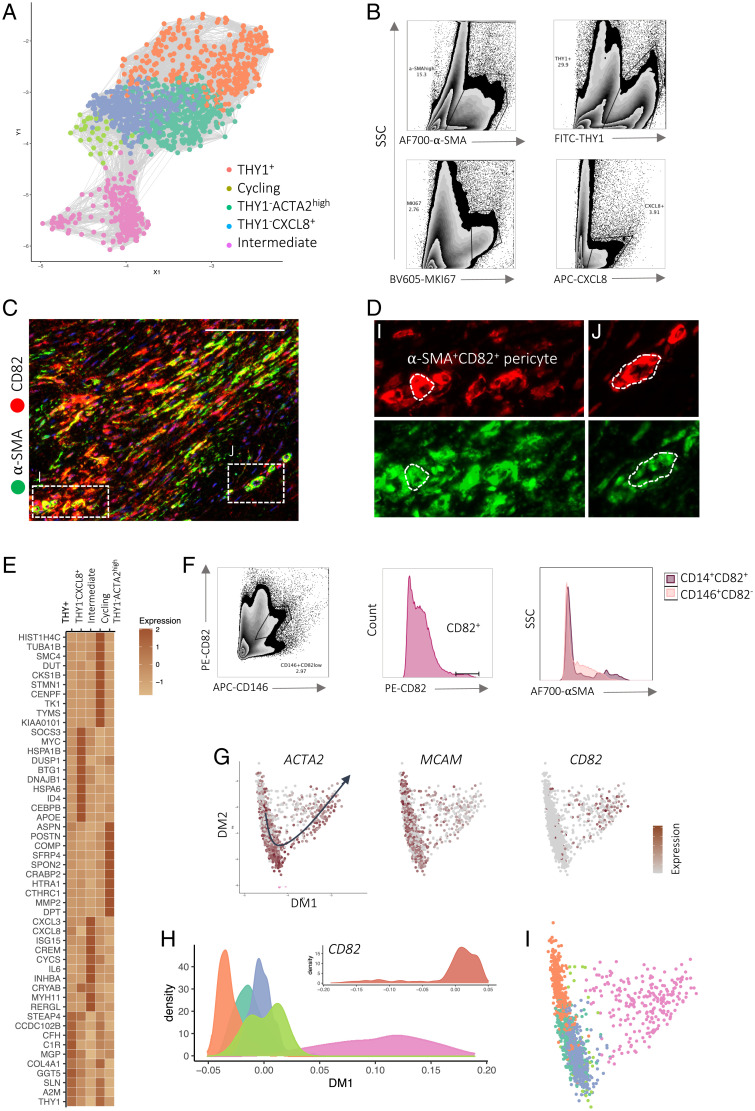
Pericytes house a putative myofibroblast precursor. (*A*) Force-directed graph (Fruchterman–Reingold layout) of scRNA-seq showing pericytes colored by major subsets (*n* = 12 DD patients). (*B*) Representative density plots of flow cytometry analysis showing pericyte (CD31^−^CD45^−^CD146^+^) subsets in freshly isolated cells from DD nodules (*n* = 8 DD patients). (*C* and *D*) Confocal images of immunofluorescence showing α-SMA^+^CD82^+^ pericytes in DD nodules (*n* = 6 DD patients). (Scale bar, 20 µm.) *D*, *i* and *j*, represent discrete sections of DD nodules. (*E*) Heatmap of scRNA-seq showing *z* score–normalized mean expression of pericyte subset marker genes (*n* = 12 DD patients). (*F*, *Left*) Density plots of flow cytometry analysis for freshly isolated nodular cells showing CD146 (MCAM) protein expression with myofibroblast marker CD82 (*n* = 8 DD patients). (*F*, *Right*) Histograms showing CD82^high^CD146^+^ pericyte and α-SMA expression in CD82^+^ and CD82^−^CD146^+^ pericytes. (*G*) Diffusion map embedding of pericytes colored by the expression of myofibroblast (*CD82*) and pericyte (*PLA2G2A*) marker genes in scaled(log(UMI + 1)). The arrow shows the principal curve fit along the first two diffusion coordinates that captured a trajectory (pseudotime) moving toward the intermediate subset from the CD82^−^ pericyte population. (*H*) Density plot of pseudotime analysis of pericyte scRNA-seq illustrating subsets and mean CD82 expression along pseudotime (DM1). Data represent Gaussian kernel probability densities. (*I*) Diffusion map embedding of scRNA-seq showing pericytes colored by major subsets.

We next examined protein and mRNA signatures of pericytes using our paired single-cell CyTOF and scRNA-seq datasets. We performed a focused analysis of pericytes by subsampling this cell type from the entire vascular dataset and repeated the analysis workflow used to define major cell classes (Fig. 2*A* and *SI Appendix*, Fig. S5). Graph-based clustering elucidated five major pericyte subsets: THY1^+^, THY1^−^CXCL8^+^, THY1^−^ACTA2^high^, cycling (MKI67^high^), and intermediate (ACTA2^high^CD82^+^) pericytes (Fig. 2 *A*, *B*, and *D*). Distinct subpopulations were validated using flow cytometry of freshly isolated nodular cells (Fig. 2*B*). Functional annotation of pericyte subset marker genes using gene ontology (GO) revealed a diversification of cellular pathways between pericyte subsets. THY1^+^ pericytes were enriched for “matrix remodeling” pathways driven by the expression of several matrix protein transcripts, including *COL1A1*, *COL6A3*, and *COL6A1* (*SI Appendix*, Fig. S5*B*). In contrast, THY1^−^CXCL8^+^ pericyte-related pathways included “endoplasmic reticulum processing,” “protein signaling,” as well as “immune activation.” Finally, THY1^−^ACTA2^high^ represented a smaller population with high expression of transcripts encoding cytoskeletal proteins such as *ACTA2*, *MYL9*, and *MYL11*, suggesting a contractile phenotype. Interestingly, markers of the intermediate pericyte showed expression of pericyte markers such as *CD146* in addition to the myofibroblast marker *CD82* (*SI Appendix*, Fig. S5*D*). Our stringent analysis workflow excluded a potential doublet cell type and we noted this subset was present across patient samples (Fig. 2*F* and *SI Appendix*, Fig. S5*A*). We verified a small population of CD82^+^CD146^+^ stromal cells using flow cytometry of freshly isolated nodular cells and in situ with multiplex immunofluorescence (Fig. 2 *C* and *D*).

To explore potential relationships between pericyte subsets, we applied diffusion maps to cells embedded in principal-component space (Fig. 2*G*). Diffusion maps are a nonlinear dimensional reduction method that provides an efficient representation of complex geometric structures and visualization of scRNA-seq data (16). This uncovered a complex topography with discrete trajectories moving to the intermediate pericyte (Fig. 2*G*). In addition, we noted this trajectory was also prominent in the principal-component space moving to the intermediate pericyte (*SI Appendix*, Fig. S6 *A*–*C*). Subsequently, we performed pseudotime analysis by fitting a principal curve across the diffusion map coordinates which allowed us to assemble gene signatures along trajectories between pericyte subsets (17). Along this modeled trajectory, we observed a coordinated decrease in pericyte markers (e.g., *MCAM*) and a gradual increase in myofibroblast markers (e.g., *CD82*), suggesting it may capture a differentiation program between pericytes and myofibroblasts (*SI Appendix*, Fig. S6*D*). After this, we examined a previously published scRNA-seq dataset containing all cell types in DD nodules to gain further perspective on the relationship between pericytes and other stromal cell types. Principal-component analysis (PCA) of this dataset again captured a distinct trajectory moving from pericytes to fibroblasts, ending at myofibroblasts (*SI Appendix*, Fig. S6 *E* and *F*). Together, pseudotemporal ordering suggested the CD82^+^CD146^+^ subset housed within profibrotic pericytes may be a myofibroblast precursor, representing an intermediate cell state along a differentiation path from pericytes to myofibroblasts.

After defining pericyte subsets in DD, we leveraged the power of this dataset to explore pericytes across a spectrum of diseases (human cirrhosis and inflammatory skin disorders, psoriasis, and atopic dermatitis [AD]) (*SI Appendix*, Fig. S5 *E*–*G*) (18). First, we performed the same computational regime to define cell subsets and constructed an integrated pericyte disease atlas comprising cells from DD, liver, and skin. Remarkably, using DD as a reference provided improved granularity for the data on human skin to expand on populations described previously (pericytes 1 and 2). We discovered THY1^+^, THY1^−^ACTA2^high^, and CXCL8^+^ pericytes in AD and psoriasis, mirroring those in DD. In contrast, in cirrhosis, THY1^+^ and THY1^−^ACTA2^high^ were both present, without a distinct CXCL8^+^ subset. Collectively, these findings suggest conserved pericyte subsets within diverse tissues undergoing inflammation and fibrosis.

### Primed Endothelial Cells Are Chemokine Centers.

**Fig. 3.**
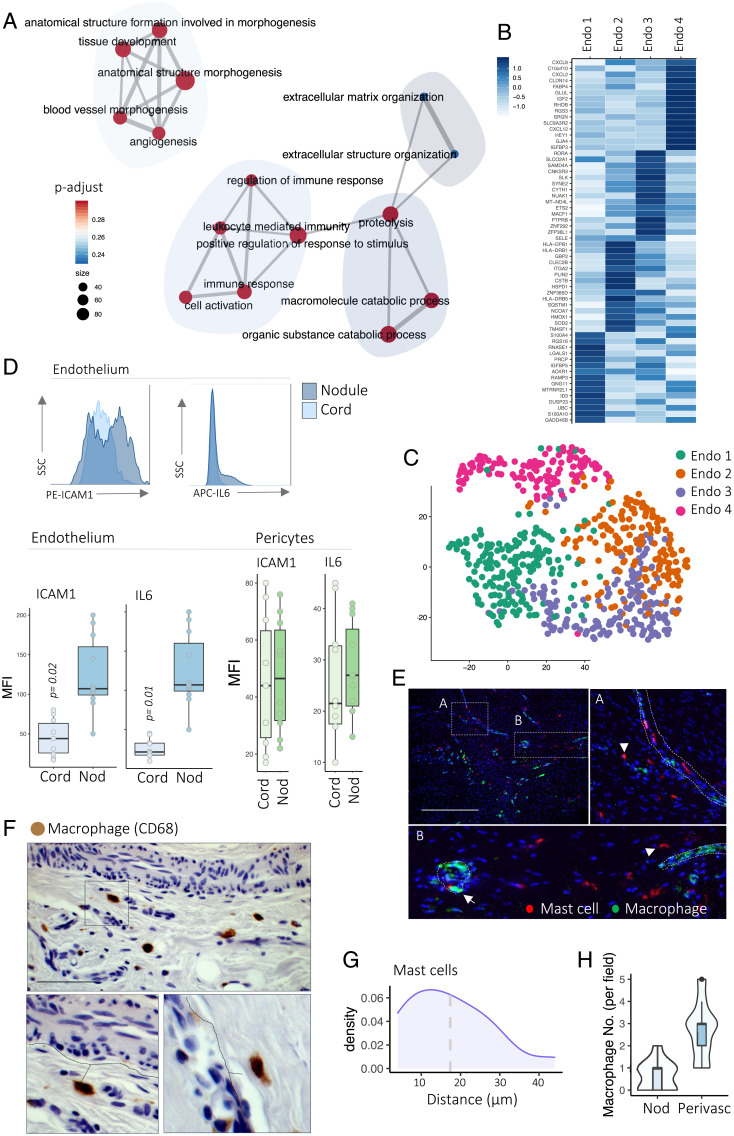
Primed endothelial cells are chemokine centers. (*A*) Network plot showing pathways enriched in endothelial cells in scRNA-seq. Gene ratio is the number of genes found in pathways; p-adjust, adjusted *P* value (two-sided Wilcoxon rank-sum test, BH FDR correction). (*B*) Heatmap of scRNA-seq showing *z* score–normalized mean expression of endothelial subset marker genes (*n* = 12 DD patients). (*C*) tSNE projections of scRNA-seq of pericytes colored by cell subsets (*n* = 12 DD patients). (*D*) Histograms (*Top*) and box and whisker plots (*Bottom*) of flow cytometry analysis showing ICAM1 and IL6 protein expression (mean fluorescence intensity [MFI]) in endothelial cells (CD31^+^) and pericytes (CD31^−^CD146^+^) in DD nodules and cords. Two-sided unpaired *t* test, mean ± SEM (*n* = 8 DD patients). (*E*) Confocal images of immunofluorescence showing mast cells (tryptase) and macrophages (CD68) in DD nodules (*n* = 6 DD patients). (Scale bar, 40 µm.) Arrowheads indicate individual mast cells in DD nodules. (*F*) Microscopy images of immunohistochemistry showing macrophages (CD68) in DD nodules (*n* = 6 DD patients). (Scale bar, 20 µm.) (*G*) Density plot of immunofluorescence analysis showing distribution of mast cell distance from vessels in DD nodules. Mean is 18.1 μm (*n* = 8 DD patients). (*H*) Violin plots of immunohistochemistry analysis showing distribution of macrophage distance from vessels in DD nodules (*n* = 8 DD patients).

Next, we focused on fibrotic endothelial cells and performed functional annotation of endothelial cell marker genes using GO. This uncovered associated endothelium pathways such as “matrix organization,” “tissue development,” and “angiogenesis” (Fig. 3*A*). A number of “inflammatory” and “immune responsive” pathways were also enriched in the endothelium, driven by the expression of immune modulators such as *IL6*, *IL8*, and *ICAM1.* Application of graph-based clustering to the endothelium defined four major subsets we termed endothelial 1 (*LGALS1^+^PRCP^+^*), endothelial 2 (*TM4SF1*^+^*ITGA2*^+^), endothelial 3 (*SELE*^+^*SAMD4A*^+^), and endothelial 4 (*CXCL8*^+^*FABP4*^+^) (Fig. 3*B*). Endothelial 4 expressed genes involved in immune pathways, including *IL6* and *IL8*. Overall, these data demonstrated transcriptomically distinct endothelial subsets in fibrosis with one subset expressing genes involved in inflammation and immune cell recruitment.

We hypothesized that the fibrotic endothelium may be primed to adopt the immunomodulatory signature demonstrated in the scRNA-seq. Inflammation is crucial in the promotion of fibrosis, and we have previously demonstrated the key role of the immune cell infiltrate in DD nodules (14, 19). To assess this, we used flow cytometry to determine endothelial expression of the immune modulators IL6 and ICAM1 within two distinct Dupuytren’s structures, the myofibroblast and immune cell–rich nodule, and the later-disease-state matrix-rich cord (Fig. 3*D*). This analysis demonstrated a higher expression of IL6 and ICAM1 in nodular endothelial cells compared with cords (Fig. 3*D*). This confirmed the endothelium shows dynamic expression of inflammatory mediators across early and late stages of human fibrosis. In contrast, pericyte expression of IL6 and ICAM1 was stable in DD nodules and cords (Fig. 3*D*).

Having delineated the dynamic expression of immune mediators in the fibrotic endothelium, we next sought to determine the spatial distribution of immune cells in DD nodules. Based on our previous finding that macrophages and mast cells are predominant sources of tumor necrosis factor (TNF) in DD (19), a central pathogenic cytokine (5, 14), we focused on these populations. Multiplex immunofluorescence microscopy and immunohistochemistry of endothelial cells (CD31), mast cells (tryptase), and macrophages (CD68) revealed a close association among these cell types (Fig. 3 *E*–*H*). Interestingly, abundant macrophages were positioned adjacent to vessels, and image analysis revealed a statistically significant relationship between endothelium, mast cells, and macrophages. Together, these data show the fibrotic endothelium has an immune modulatory signature with up-regulated expression of ICAM1 and IL6 and show a close spatial association with immune cells in localized human fibrosis.

### Coupling of the Fibrotic Vasculature.

After this, we used systematic analysis of receptor–ligand interactions to predict pathways mediating cell-to-cell communication (*SI Appendix*, Fig. S7 *A*–*D*). Given their close anatomical approximation, we first focused on interactions between pericytes, endothelial cells, and immune cells (*SI Appendix*, Fig. S7*B*). Several statistically significant paracrine and autocrine interactions were distinguished between ligands and cognate receptors expressed by pericytes and endothelial cells, including *IL6*-*IL6ST*, *PDGF*-*PDGFR*, and *NOTCH3-JAG1* (*SI Appendix*, Fig. S7 *B* and *C*). Pericytes expressed numerous integrin receptors (*ITGB1*, *ITGAV*, and *ITGB2*) that are known to bind collagens in the vessel wall. In addition, pericytes showed specific expression of *NOTCH3* and its cognate receptor *JAG1* on endothelial cells. *NOTCH3* expression was localized to THY1^+^ pericytes and *JAG1* to the CXCL8^high^ endothelial 4 populations (*SI Appendix*, Fig. S7*B*). In addition, in the scRNA-seq data, we noted collagen VI was a common signaling partner in endothelial cell and pericyte interactions (*SI Appendix*, Fig. S7*F*). We therefore sought to validate the expression of putative cell type–specific signaling partners. Using multiplex immunofluorescence, we confirmed NOTCH3 and collagen VI expression in fibrotic perivascular cells. NOTCH3^+^ pericytes were readily identified within vessels in DD nodules and NOTCH3 expression was restricted to pericytes (*SI Appendix*, Fig. S7*E*). In contrast, we observed high expression of collagen VI in pericytes and myofibroblasts in DD nodules (*SI Appendix*, Fig. S7*H*). Projecting COL6A1 expression across the DD vasculature scRNA-seq dataset demonstrated high mRNA expression specifically in pericytes, confirming COL6A1 as a specific marker which can discriminate pericytes from endothelial cells in DD nodules (*SI Appendix*, Fig. S7*G*).

### Endothelial Cells Sustain Fibroblast Identity.

**Fig. 4.**
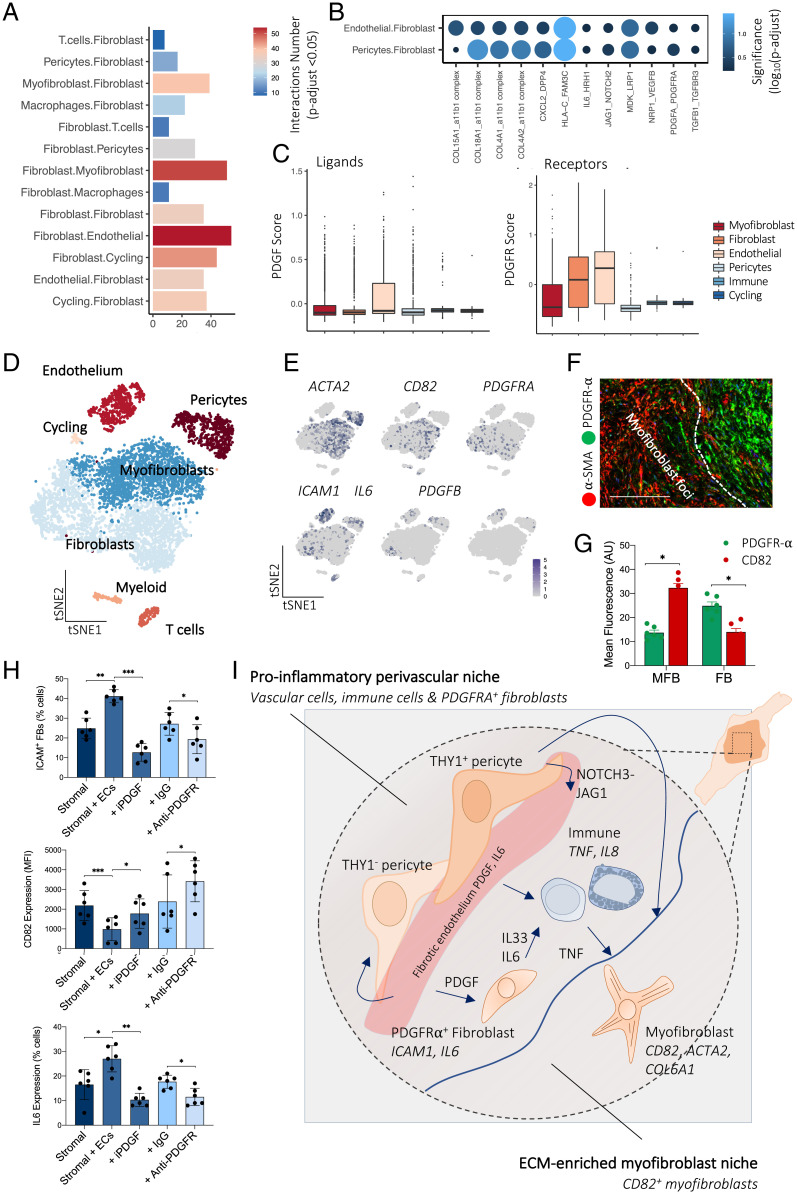
Endothelial cells sustain fibroblast phenotype. (*A*) Barplot showing the number of significant interactions between cell types in DD nodules defined by CellPhoneDB analysis of scRNA-seq (*n* = 12 DD patients). Adjusted *P* < 0.05 was defined as significant (two-sided Wilcoxon rank-sum test, BH FDR correction). (*B*) Dotplot showing significance (color and size) of receptor–ligand pairs in significantly enriched pathways following interaction analysis of scRNA-seq. Two-sided Wilcoxon rank-sum test, BH FDR correction. (*C*) Box and whisker plots showing PDGF ligand and receptor gene module expression in DD cell types defined in scRNA-seq (*n* = 12 DD patients). (*D*) tSNE projection of scRNA-seq in entire DD nodules showing major cell types (*n* = 12 DD patients). (*E*) tSNE projections of scRNA-seq showing RNA expression (log(UMI + 1)) in DD cells of selected genes characterizing major cell populations. (*F*) Confocal images of immunofluorescence showing PDFGR-α expression in fibroblasts and CD82 in myofibroblasts within DD nodules (*n* = 3 DD patients). (Scale bar, 40 µm.) (*G*) Barplot showing quantitative analysis (mean fluorescence; arbitrary unit, AU) of immunofluorescence microscopy of PDFGR-α and CD82 expression in DD nodules. MFB represents myofibroblast foci and FB represents the fibroblast niche in DD nodules, two-sided unpaired *t* test, mean ± SEM (*n* = 6 DD patients). *P < 0.05. (*H*) Barplots of flow cytometry analysis of DD and endothelial cell coculture assay. iPDGF represents crenolanib and anti-PDGFR represents the anti–PDGFR-α/β antibody mixture. Two-sided unpaired *t* test, mean ± SEM (*n* = 6 DD patients). *P < 0.05; **P < 0.01; ***P < 0.001. (*I*) Schematic illustrating stromal–immune cell cross-talk in DD. Immune cells (mast cells and M2 macrophages) are the predominant source of TNF that leads to the development of the myofibroblast phenotype. Myofibroblasts are a significant source of IL33, which further activates TNF secretion from immune cells. The endothelium acts to promote formation of fibroblasts.

Based on the location of vessels outside the myofibroblast foci, we hypothesized that the vasculature may act to sustain the perivascular PDGFR-α^+^ fibroblast identity. Receptor–ligand analysis again demonstrated myriad significant intercellular signaling pathways between the endothelium and fibroblasts, including PDGFB–PDGFR, collagen–integrin, and IL6–IL6ST pathways. Interestingly, we noted the highest number of significant interactions between fibroblasts and endothelial cells, suggesting a close functional association (Fig. 4 *A* and *B*). After this, we examined central signaling pathways between fibroblasts and endothelial cells. Constructing PDGFR and PDGF gene modules using the average expression of PDGF receptors and ligands uncovered endothelial cells as the top cell type enriched for PDGF ligands and fibroblasts and endothelial cells as the top cell types enriched for PDGF receptors (Fig. 4*C*). Together, these transcriptomic data suggest PDGF signaling may coordinate the interaction between fibroblasts and endothelial cells in fibrosis.

We then tested whether endothelial cells could directly sustain fibroblast identity given their proximity in vivo. In DD, we have previously shown that stromal cells exist along a functionally distinct continuum between ICAM1^+^IL6^high^ fibroblasts (PDGFR-α^+^) and CD82^+^ myofibroblasts (15) (Fig. 4 *D* and *E*). We applied a two-dimensional hydrogel system that supports the stable coculture of DD stromal cells and endothelial cells in a mechanically aware environment (*SI Appendix*, Fig. S8). Seeding freshly isolated DD stromal cells with or without endothelial cells, we measured the expression of fibroblast (*ICAM1* and *IL6*) and myofibroblast (*CD82*) markers. Consistent with endothelial cells as a source of differentiation cues for perivascular fibroblasts, we observed a down-regulation of myofibroblast differentiation marker CD82 and up-regulation of ICAM1^+^ on coculturing stromal cells with endothelial cells (Fig. 4*H*). Next, we sought to determine whether PDGF signaling enabled this interaction between endothelial cells and DD stromal cells. First, we noted specific expression of PDGFR-α in the fibroblast compartment as quantified by immunofluorescence staining of DD nodules (Fig. 4 *F* and *G*). Following this, we performed the hydrogel coculture assay with the addition of crenolanib, a small molecular PDGF inhibitor. This inhibited the fibroblast phenotype (ICAM1^+^IL6^+^) and increased expression of CD82 (Fig. 4*H*). We confirmed these results with antibodies targeting PDGFR-α/β in the coculture system (Fig. 4*H*). Taken together, these results demonstrate endothelial cells directly modulate fibrotic stromal cell differentiation and, via PDGF signaling, act to sustain an immunomodulatory perivascular ICAM1^+^IL6^high^ fibroblast identity.

## Discussion

We combined scRNA-seq, CyTOF, and in situ tissue imaging to build a spatially resolved cell atlas of the fibrotic vasculature. Our study of patients with localized fibrosis in DD nodules revealed discrete populations of perivascular and endothelial cells based on protein and mRNA profiles. We observed an endothelial subset expressing an immunomodulatory program and demonstrated across early and late stages of fibrosis that the endothelium exhibits dynamic expression of immune modulators such as ICAM1 and IL6. In contrast, pericytes did not share this signature, suggesting a discrete function of the endothelium to maintain immune cell recruitment. Supporting this in DD nodules the endothelium, mast cells, and macrophages occupied a perivascular niche.

Understanding the origin and activation of matrix-producing myofibroblasts is essential to develop targeted therapeutics in fibrosis, and this perspective is currently lacking in many human fibrotic diseases. Our findings suggest pericytes may provide a pool of myofibroblasts in DD and we demonstrated a cell subset in an intermediate pericyte–myofibroblast state. Previous studies have revealed pericytes as myofibroblast precursors in visceral fibrosis in the lung (20, 21) and kidney (5, 22). In addition, pericytes have been shown to adopt myofibroblast properties in the fibrotic lung microenvironment (23). These data suggest that pericytes may be precursors of collagen-producing myofibroblasts across distinct fibrotic systems, and our results support this phenomenon in localized human fibrosis. Crucially, pseudotime analysis positioned the CD82^+^CD146^+^ intermediate pericyte along a modeled trajectory associated with a myofibroblast differentiation program. Given the lack of an animal model for DD, we are unable to directly compare our findings in an in vivo model, including potential functional and lineage tracing studies of stromal cell populations. We were also unable to compare our findings from early-stage DD with a normal, healthy control, as the unaffected palmar fascia comprises a densely packed extracellular matrix with very few cells (24), and we have previously shown that multiple passaged cells change their phenotype (25). Moving forward, integrating the transcriptomic program marking the pericyte-to-myofibroblast transition in different organs will help to elucidate central regulators of this process.

Identification of the intermediate pericyte in DD may help to resolve analogous cell states in visceral human fibrosis. Interestingly, a recent scRNA-seq study in renal fibrosis resolved a differentiation path from NOTCH3^+^ pericytes to myofibroblasts suggesting this subset may be a conserved precursor across diverse fibrotic disorders (5). Our integrative analysis of skin, liver, and DD fibrosis captured shared pathological pericyte phenotypes that may be universal to inflammatory disease. THY1^+^ and THY1^+^ACTA2^+^ pericytes were identified in all organs, in contrast to the intermediate or CXCL8^+^ subsets absent in cirrhotic liver. Many factors likely drive this cell-type variation across organs, including histological architecture, tissue mechanics, and local cellular niches. Indeed, the high expression of many mRNA-encoding cytoskeleton proteins in pericytes and the division of cell subsets by those encoding contractile proteins (*ACTA2*, *MYL9*, and *MYLK*) suggest mechanical stress and tissue mechanics may contribute to the development of distinct pericyte subsets.

Topological organization in the human body facilitates effective segregation and integration of cellular function (Fig. 4*I*) (26). Therefore, it is reasonable that pathological systems may have conserved functional and structural organization to sustain the disease state. Indeed, a recent study in rheumatoid arthritis confirmed that patterning of stromal cells in the inflamed joint allowed cell types to adopt diverse functions (27). Discoveries such as this are still lacking in fibrosis. Here, integrating the transcriptomic and spatial context of cells at high resolution demonstrated the vasculature occupied a distinct anatomical niche. Blood vessels resided in close approximation to PDGFR-α^+^ fibroblasts, suggesting intercellular communication between these lineages, supported by cell–cell interaction analysis. Moreover, within the perivascular niche we observed an enrichment of immune cells, proposing this compartment has a central role in sustaining inflammation. We also demonstrated analogous stromal cell compartmentalization in the hand and lung, confirming distinct myofibroblast foci and fibroblast niches in IPF and DD. Despite conserved markers of stromal cells (PDGFR-α and α-SMA), myofibroblastic focus morphology was more heterogeneous in the lung than the hand palmar fascia, which may relate to the early-stage DD and the late-stage IPF tissue samples.

More generally, leveraging the power of a complete fibrotic ecosystem in DD, we show a global organization of cellular lineages in human fibrosis encompassing distinct myofibroblast and fibroblast compartments. Within visceral fibrosis of the lung (28) and liver (29), myofibroblast compartments have been described previously, but our results resolve a separate immunomodulatory PDGFR-α^+^ fibroblast-enriched perivascular niche. In addition, most significantly, using primary human coculture systems, we highlight the endothelium as not merely a bystander in fibrosis but instead acts to support the differentiation of perivascular immune regulatory fibroblasts through PDGF signaling. ICAM1^+^ fibroblasts have been demonstrated to be enriched in developing fibrosis in DD and function to promote immune cell recruitment (15). Therefore, by up-regulating this population the endothelium may be a central regulator of inflammation as well as by the direct expression of immune modulators (e.g., ICAM1 and IL6).

It is well established in many disorders that inflammation triggers stromal cell activation and subsequent matrix deposition in fibrosis. Hence, identifying its role in promoting inflammation and residency during early and late stages of disease suggests the endothelium may be pivotal in triggering the nascent stages of fibrosis. Furthermore, the expression of several human leucocyte antigen (HLA) molecules (HLA-DPB1, HLA-DRB1, and HLA-DRB5) in endothelial cells indicates that an autoimmune mechanism may stimulate their inflammation phenotype in DD. However, despite the clear association with specific HLA alleles (30) and predilection toward or protection from DD, a confirmed autoimmune driver in DD is still lacking. The presence of antiendothelial autoantibodies in models of systemic sclerosis highlights how vasculopathy (31) can drive localized fibrosis in the skin, but a serum autoantibody has not been identified in DD. Finally, by controlling the equilibrium between fibroblast and myofibroblast formation, the endothelium may also influence global functions of profibrotic stroma and coordinate the precise geometrical positioning of pathologically distinct stromal cell subsets (15).

In summary, our results demonstrate a vascular niche in human fibrosis that orchestrates stromal cell identity, promoting fibroblast differentiation while inhibiting myofibroblast formation, thereby governing topological organization in the fibrotic milieu. Beyond this, our results build a single-cell atlas of localized human fibrosis and show how the endothelium instructs the differentiation of functionally distinct stromal cells spanning between collagen-producing contractile CD82^+^ myofibroblasts and immunomodulatory ICAM1^+^ fibroblasts. While our results are mainly derived from DD, the underlying mechanisms we describe also appear to be relevant to other important fibrotic diseases such as IPF, where samples of early-stage disease are more difficult to access. The potential for targeting vascular pathways in human fibrosis is highlighted by the recent approval of the tyrosine kinase inhibitor nintedanib for IPF (32), which in part acts by down-regulating PDGF signaling.

## Methods

### Patient Samples and Cell Culture.

After approval by the local ethical review committee (REC 07/H0706/81), tissue samples were obtained with informed consent from patients with DD. Patient samples were deidentified prior to use in the study. Dupuytren’s nodular tissue was obtained from individuals with DD undergoing dermofasciectomy (15, 19). Cells from DD were isolated from α-SMA–rich nodules as described previously (25, 33). Tissue samples were dissected into small pieces and digested in Dulbecco’s modified Eagle’s medium (DMEM) (Lonza) with type I collagenase (Worthington Biochemical) + DNase I (Roche Diagnostics). Cells were cultured in DMEM with 5% (volume/volume; vol/vol) fetal bovine serum (FBS) and 1% penicillin–streptomycin at 37 °C in a humidified incubator with 5% (vol/vol) CO_2_. Cells before passage 2 were used for experiments and only freshly isolated cells were used for scRNA-seq, CyTOF, and flow cytometry.

### Antibodies.

Antibodies used in flow cytometry are as follows: PE-ICAM1 (BioLegend; HA58), PE/Cy7-CD34 (BioLegend; 581-BL), APC-PDPN (BioLegend; NC-08), APC-CD31 (BioLegend; WM59), APC-CD146 (BioLegend; SHM-57), FITC-CD146 (Abcam; PIHI2), BV421-CD45 (BioLegend; HI30), BV605-ki67 (BioLegend; Ki67), APC-IL8 (eBioscience; BCH), APC-IL6 (BioLegend; MQ2-13A5), AF700-α-SMA (R&D Systems; ICI420N), APC-CD29 (BioLegend; 152/16), and PE-CD82 (BioLegend; ASL-24). Antibodies used for immunohistochemistry and immunofluorescence are as follows: α-SMA (Abcam; A5228), PDGFRA (Novus Biologicals; NBP2-76508), CD31 (Abcam; ab24590), tryptase (Abcam; ab2378), CD68 (Abcam; ab213363), and CD82 (Abcam; TS82b).

### Single-Cell Isolation.

DD samples were rapidly transported to the research facility following surgical resection. Dupuytren’s nodules were minced using a scalpel and transferred to 5 mL of digestion medium described above. Dissociated cells were then washed in 5% FBS in DMEM (Gibco) and passed through a 100-μm cell strainer. This single-cell solution was slowly frozen using a Mr. Frosty container (Thermo Fisher). Single-cell suspensions were thawed on the day of sequencing, and diluted at a concentration of 1,000 cells per microliter in 0.04% bovine serum albumin (BSA) in phosphate-buffered saline (PBS) for loading into 10X Chromium Single Cell A chips.

### Droplet-Based scRNA-Seq.

Single-cell libraries were prepared using the Chromium 3′ v2 platform (10X Genomics) following the manufacturer’s protocol (15, 19). In brief, single cells were encapsulated into gel beads in emulsions (GEMs) in the GemCode instrument followed by cell lysis and barcoded reverse transcription of RNA, amplification, shearing, and 3′ adaptor and sample index attachment. Approximately 10,000 single cells were loaded per channel and ∼1,800 to 6,500 cells were sequenced. Libraries were sequenced on the Illumina HiSeq 4000 (paired-end reads: read 1, 26 bp; read 2, 98 bp).

### Computational Analysis.

Sample demultiplexing, alignment to the GRCh38 human transcriptome, and unique molecular identifier (UMI) collapsing were performed using the Cell Ranger toolkit (v2; 10X Genomics). Core steps in the downstream analysis were undertaken using the Seurat R package (Sajita Lab, New York Genome Centre). Quality control was performed on each patient sample separately. We excluded poor-quality cells that expressed fewer than 200 genes and with over 10% of UMIs mapping to mitochondrial genes as well as cells that expressed over 4,500 genes. Batch correction was performed using Combat as implemented in the sva R package using the default parametric adjustment mode (34). Batch correction was assessed before downstream analysis and we observed minimal batch effects following the Combat method. We employed a global-scaling normalization procedure as per ref. 35 calling Seurat’s LogNormalize() function. For this, the Cell Ranger UMI count value for each gene was divided by the sum of the total UMI counts per cell to normalize for differences in library size and then multiplied by a scaling factor that represented the median library complexity (10,000) producing transcripts per million-like values. We then took the log transform of this procedure for downstream analysis. Downstream analysis was performed using all patient donors (*n* = 12). Feature selection was first undertaken by defining highly variable genes using the FindVariableGenes() function (1,566 genes). Values were then centered and scaled before input to PCA, which was implemented using the R function prcomp from the stats R package (15, 19). After PCA, significant PCs were identified using the permutation test implemented using the permutationPA function from the jackstraw R package (36). This test identified 22 significant PCs and these were used as the input to further analysis.

To group vascular cells, we applied unsupervised clustering based on the Louvain algorithm with the Jaccard correction implemented in the FindClusters() function. Vascular cell clusters were then annotated to known biological cell types using canonical marker genes. Regression of the cell-cycle effect did not significantly influence clustering and therefore we opted not to undertake correction for cell-cycle effects. We then subsampled each (pericytes and endothelial cells) cell type to define putative subclusters. We applied the same steps as described above to delineate subclusters including selection of highly variable genes, PCA, and selection of significant PCs. As the average UMI count varied between distinct cell types when analyzing individual cell types, we performed a second round of quality control to remove doubles and contaminants. We used a set of canonical markers for each cell type and excluded those cells that shared markers of multiple cell types and those cells with a UMI count 3 SDs above the median cell-type UMI count. For visualization, we used the Barnes–Hut approximate version of *t*-distributed stochastic neighbor embedding (tSNE) using the Rtsne R package.

On inspection of the PC projection of the pericyte dataset, we noted a distinct trajectory moving to the intermediate population associated between *MCAM* and CD82 marker expression. *CD82* is an established myofibroblast marker and *MCAM* is a pericyte marker. This trajectory was apparent in the pooled datasets and individual patient samples. We therefore reasoned that this may represent the transcriptional profile of a differentiation pathway. To allocate each pericyte subtype along the putative activation trajectory, we first performed dimensional reduction using diffusion maps, implemented in the R package destiny (16). The distance metric was the Euclidean distance between pairs of cells in the reduced dimension space of the significant PCs (*n* = 22). In concordance with the PCA, the first two diffusion components were highly associated with *CD82* expression, capturing a trajectory moving through the pericyte subtypes to the intermediate population. We then fitted a principal curve (R package princurve, smoother = lowess, *f* = 1/3) through the first two diffusion coordinates (37). As the λ value of the curve reflects the arc length from the beginning of the curve for each point, we used this to assign each cell to a “pseudotime” trajectory. Expression smoothing was performed using generalized additive models implemented in the gam function in the mgcv R package. This modeled trajectory was focused on capturing differentiation associated with *CD82* expression but did not ascribe any distinct cell type as the myofibroblast precursor. In addition to this approach, we also used the slingshot R package to infer trajectories and align cells along developmental pseudotime.

GO enrichment of cluster markers and differentially expressed genes was performed using the R package clusterProfiler (38) with a Benjamini–Hochberg (BH) multiple testing adjustment and a false-discovery rate (FDR) cutoff of 0.1, using all expressed genes expressed in >3 cells as background. Visualization was performed using the R packages ggplot2 and igraph (15, 19).

For comprehensive systematic analysis of interlineage interactions, we used CellPhoneDB (39). In brief, we derived putative ligand–receptor interactions on the expression of a receptor and its cognate ligand across lineage subpopulations. We considered only ligands and receptors expressed in greater than 5% of the cells in any given subpopulation. After this, for PDGF–PDGFR signaling analysis, we defined receptor and ligand gene modules in distinct cell types to prioritize central intercellular interactions mediated by this pathway. To visualize this, we obtained a gene list of the PDGF ligands and PDGF receptors and then defined the average expression of these gene modules minus a control gene set. To account for differences in library complexity between cells, we calculated a control gene set from these scores (40). This was selected by binning all genes in the dataset and randomly selected 100 genes from each bin that contained every gene of the test gene set. Thus, the control gene set has a comparable distribution of expression levels to that of the PDGF ligand and PDGF receptor gene sets and the control gene set is 100-fold larger, such that its average expression is analogous to averaging over 100 randomly selected gene sets of the same size as the PDGF ligand and PDGF receptor gene sets.

### Flow Cytometry.

Single-cell suspensions were stained with a panel of fluorescently labeled antibodies to surface antigens, washed with fluorescence-activated cell sorting wash buffer (1% BSA, 0.01% NaN_3_ in PBS), and then fixed using CytoFix (eBioscience; Foxp3 Staining Buffer Set 00-5523-00) for a minimum of 30 min at 4 °C. We performed three washes in permeabilization wash (eBioscience; Foxp3 Staining Buffer), intracellular antigens were stained with another panel of fluorescently labeled antibodies, and cells were then washed with permeabilization wash and analyzed by flow cytometry (BD; LSRFortessa X-20) and FlowJo software. BD CompBeads (anti-mouse immunoglobulin K; 552843) were used to establish compensation settings. Dead cells were deselected using live/dead stain, added to the surface staining panel prior to cell fixation (Life Technologies; Live/Dead Fixable Near-IR Dead Cell Stain Kit L10119). Isotype controls were used during antibody optimization.

### Mass Cytometry.

Antibodies not purchased from Fluidigm were labeled using metal tags using Maxpar Antibody Labeling Kits (Fluidigm). Antibody titration was undertaken and used at a concentration from 0.25 to 0.5 μg/mL. One to three million cells were first stained per sample with a solution containing rhodium DNA intercalator (Fluidigm) to distinguish live/dead cells before Fc receptor blocking (Miltenyi Biotec). Samples were then stained with the mixture of conjugated antibodies for cell-surface antigens. After washing in Maxpar Cell Staining Buffer (Fluidigm), samples were fixed and permeabilized using the Foxp3 Transcription Factor Staining Buffer Kit (Thermo Fisher) prior to washing and incubation with metal-conjugated antibodies recognizing intracellular antigens. Samples were washed twice in cell-staining buffer, fixed by incubation with 1.6% paraformaldehyde (PFA) (Pierce) for 10 min, and finally incubated overnight with iridium DNA intercalator in Maxpar Fix and Perm Buffer (Fluidigm). Samples were washed again, being acquired on a Helios mass cytometer (Fluidigm). After acquisition, .fcs files were normalized using tools within Helios software, and gating and downstream analysis using clustering and dimensionality reduction algorithm viSNE was performed using Cytobank (https://www.cytobank.org/).

### CyTOF Antibodies.

CD252 169-Tm (3166007B; ML5; Fluidigm), CD34 166-Er (3166012B; clone 581; Fluidigm), CD45 141-Pr (3141009B; clone HI30; Fluidigm), HLA-D A, B C 142-Nd (3142007B; clone HCD57; Fluidigm), CD19 143-Nd (3144007A; clone NP6G4; Fluidigm), HLA-DR 89-Y (3173005B; L243; Fluidigm), B-catenin 176-Lu (3147005A; clone D10A8; Fluidigm), CD55 174-Yb (3148015B; clone JS11; Fluidigm), CD146 155-Nd (3155006B; P1H12; Fluidigm), CD29 156-Gd (3156007B; TS2/16; Fluidigm), CD82 158Gd (3158025B; ASL-24; Fluidigm), CD90 172-Yb (3173011B; 5E10; Fluidigm), TGFb 150-Nd (3163010B; clone TW46H10; Fluidigm), Ki67 168-Er (3168007B; B56; Fluidigm), CD54 170-Er (3170014B; HA58; Fluidigm), CD68 171-Yb (3171011B; Y1/82A; Fluidigm), CD9 172-Yb (312102; BioLegend), CD252 150-Nd (326302; BioLegend), a-SMA 156-Gr (MAB1420; 1A4; R&D Systems), gp38 155-Gd (AF3670-SP; R&D Systems), PDGFRB 154-Sm (SAB4700458; 18A2; Sigma-Aldrich), and MMP14 149-Sm (MAB9181-SP; 128527; R&D Systems).

### Immunohistochemistry.

We performed fixation of Dupuytren’s tissue samples with 4% PFA in PBS for 20 min which were then embedded in paraffin wax, and 7-μm sections from the cut surface were processed for immunohistochemistry (41). Antibodies were detected using a two-staged polymer enhancer system (Sigma). Murine or rabbit IgG isotypes at the same protein concentration as the monoclonal antibody solution were used as a control. Images were acquired with an Olympus BX51 microscope.

### Immunofluorescence and Confocal Microscopy.

Dupuytren’s tissue samples were fixed with 4% PFA in PBS for 20 min, longitudinally bisected, and embedded in paraffin wax, and 7-μm sections from the cut surface were processed for immunofluorescence (41). Then, the tissue sections were stained with antibodies listed above followed by incubation with fluorescent dye–conjugated secondary antibodies (Life Technologies). Nuclei were counterstained with DAPI (Sigma-Aldrich) and mounted using ProLong Gold Antifade Mountant (Life Technologies). Fluorescent images were obtained with a confocal system (Zeiss; LSM 710).

### Hydrogel and Imaging for Coculture Assay.

Polyacrylamide (PAA) hydrogels were prepared as previously described (42). Briefly, PAA gel formation was initiated with ammonium persulfate (10% solution in ddH_2_O; Sigma-Aldrich) and *N*,*N*,*N*′,*N*′-tetramethylethylenediamine (Sigma-Aldrich). Polymerized PAA gels were functionalized with sulfo-SANPAH (Invitrogen) and coated with type 1 collagen (200 μg/mL rat tail; Thermo Fisher). The Young’s modulus of the PAA gels was 8.55 ± 0.5 kPa. Cells were allowed to adhere to the gel for 4 to 6 h before flow cytometry.

### Coculture of Endothelial Cells and DD Stromal Cells.

DD stromal cells were seeded onto PAA hydrogels with and without primary human umbilical vein endothelial cells in complete endothelial medium. Cells were cultured for 72 h in endothelial basal medium with or without the PDGF signaling inhibitor crenolanib (1 μM; Cayman Chemicals; CAY1873) or vehicle (dimethyl sulfoxide) control, in addition to an anti-PDGFRa/b antibody mixture (PDGFR-α, Invitrogen, 710169; PDGFR-β, Invitrogen, G.290.2). Cells were fixed and stained using the flow cytometry protocol detailed above.

## Supplementary Material

Supplementary File

## Data Availability

The source data for the scRNA-seq reported in this article have been deposited in the Sequence Read Archive public repository under accession number PRJNA607098. All other study data are included in the article and/or *SI Appendix*.

## Change History

April 1, 2022: The article copyright license has been updated.
