# Supplementary material for: A vasculature niche orchestrates stromal cell phenotype through PDGF signaling: Importance in human fibrotic disease
Source: Proc Natl Acad Sci U S A. 2022 Mar 23;119(13):e2120336119. doi: 10.1073/pnas.2120336119 (PMC9060460; doi:10.1073/pnas.2120336119)
Supplement: Supplementary File [file pnas.2120336119.sapp.pdf]

A vasculature niche orchestrates stromal cell phenotype through PDGF signaling: importance in human fibrotic disease

Authors
Thomas B Layton[1], Lynn Williams[1], Nan Yang[1], Mingjun Zhang[2], Carl Lee[1], Marc Feldmann[1], Glenda Trujillo[3], Dominic Furniss[4], Jagdeep Nanchahal[1†]

Affiliations
[1]The Kennedy Institute of Rheumatology, Nuffield Department of Orthopaedics, Rheumatology and Musculoskeletal Sciences, University of Oxford, UK
[2]Biotherapeutics, Bristol-Myers Squibb, San Diego, California, United States of America.
[3]Fibrosis Biology Drug Discovery, Bristol-Myers Squibb, Lawrenceville, New Jersey, United States of America.
[4]Nuffield Department of Orthopaedics, Rheumatology, and Musculoskeletal Sciences, University of Oxford, Botnar Research Centre, UK.

†Corresponding author. Email: jagdeep.nanchahal@kennedy.ox.ac.uk

One Sentence Summary: In human fibrosis a discrete vascular niche housing a myofibroblast precursor is critical in maintaining the proinflammatory microenvironment by supporting immune regulatory fibroblast identity through PDGF signalling.

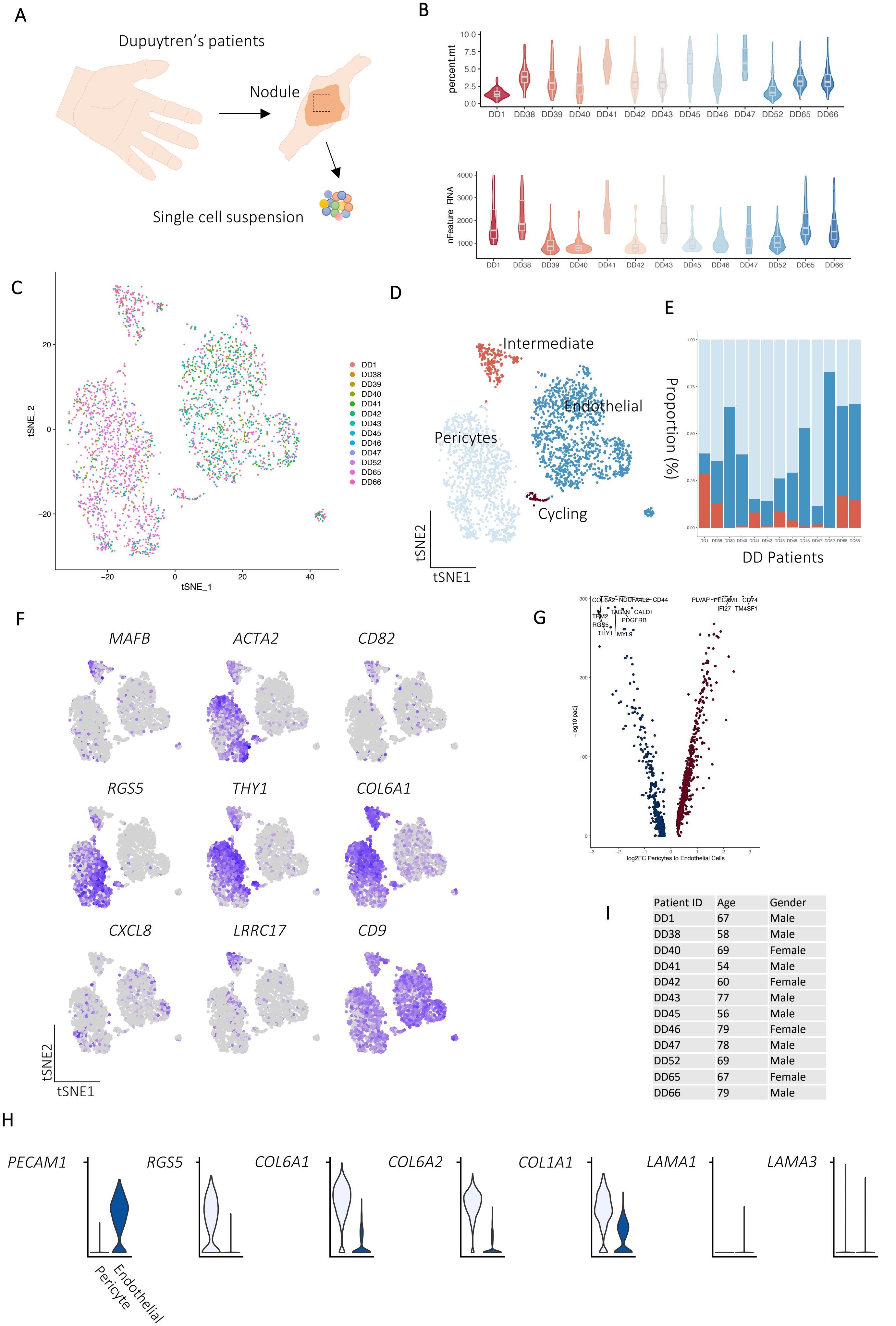

Patient ID	Age	Gender
DD1	67	Male
DD38	58	Male
DD40	69	Female
DD41	54	Male
DD42	60	Female
DD43	77	Male
DD45	56	Male
DD46	79	Female
DD47	78	Male
DD52	69	Male
DD65	67	Female
DD66	79	Male

Fig. S1. Single cell RNA-seq of Dupuytren's nodules.

(A) Schematic illustrating experimental protocol for single cell profiling of Dupuytren's nodule. (B) Violin plots showing quality control metrics in DD patients (in single cell RNA-seq. Y axis in log(UMI+1) for nUMI and percentage of cells for mitochondrial reads. DD is prefix for DD patient IDs (*n* = 12 DD patients). (C) tSNE projections showing patient donors for 7,332 cells (*n* = 12 DD patients) in single cell RNA-seq. (D) tSNE projection showing major cell types for 7,332 cells (*n* = 12 DD patients) in single cell RNA-seq. (E) Stacked bar plot showing the proportion of pericytes, intermediate pericytes and endothelial cells in each Dupuytren's nodule (*n* = 12 DD patients, DD is prefix for patient ID. (F) tSNE projections showing expression in log(UMI+1) of selected genes for major cell types for 7,332 cells (*n* = 12 DD patients) in single cell RNA-seq. (G) Volcano plot showing differentially expressed genes (2-sided Wilcoxon Rank Sum Test) between pericytes and endothelial cells in single cell RNA-seq with FDR corrected adjusted *p*-values (BH correction). (H) Violin plots showing normalized mRNA expression of matrix proteins in endothelial cells and pericytes. (I) Table showing patient ID and details for 12 patients in scRNA-seq data.

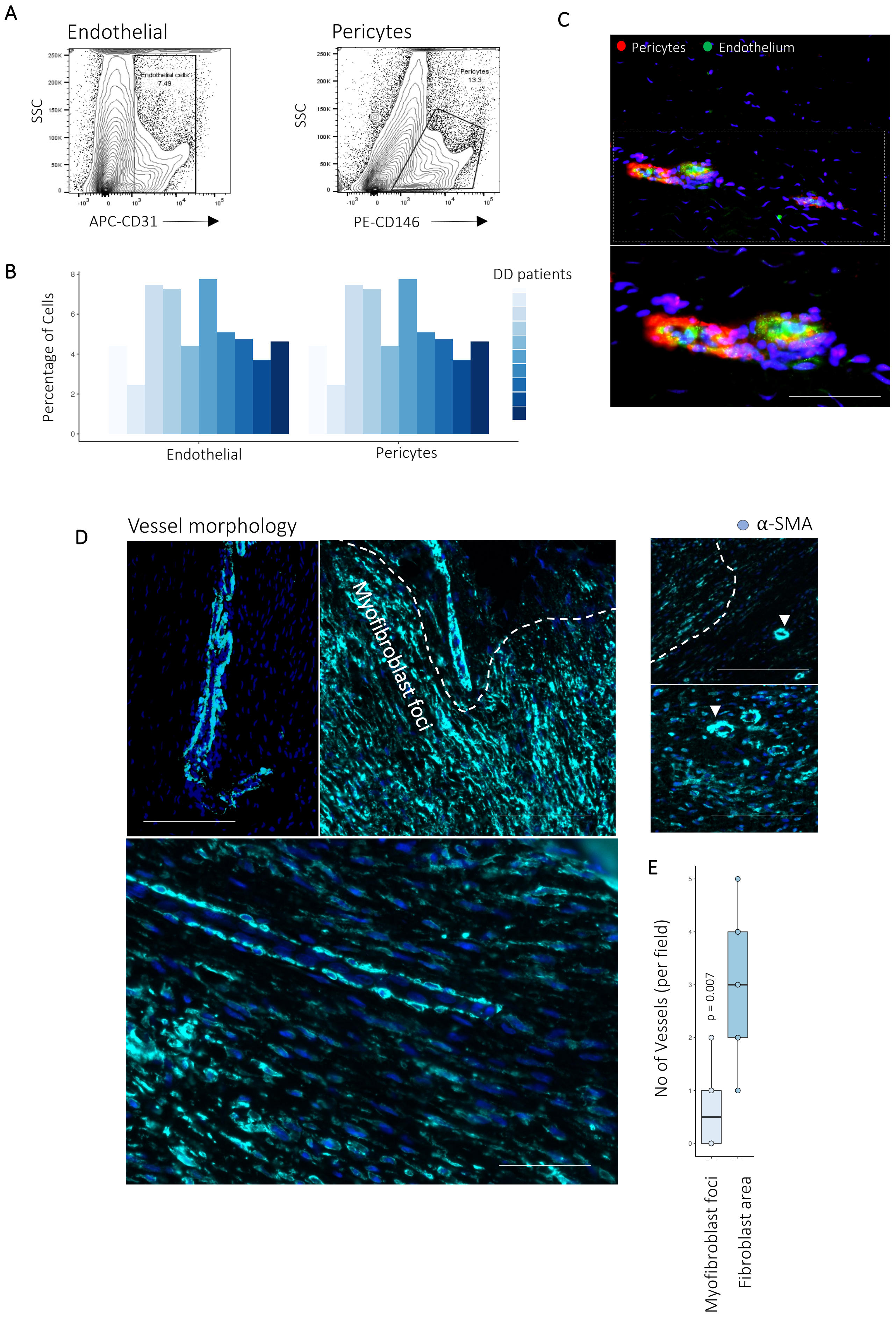

A
Endothelial
Endothelial cells 7.49
SSC
APC-CD31
Pericytes
Pericytes 13.3
SSC
PE-CD146
B
Percentage of Cells
DD patients
Endothelial
Pericytes
C
Pericytes
Endothelium
D
Vessel morphology
α-SMA
Myofibroblast foci
E
No of Vessels (per field)
p = 0.007
Myofibroblast foci
Fibroblast area

Fig. S2. Validation of a vascular compartment in DD

(A) Representative density plots of flow cytometry analysis showing endothelial cells ($CD31^+$) and pericytes ($CD146^+$) in freshly isolated DD nodular cells ($CD45^-$) (n = 6 DD patients). (B) Bar plots of flow cytometry analysis in freshly isolated DD nodular cells ($CD45^-$) showing the proportion of total cells isolated as pericytes and endothelial cells. (C) Confocal images of immunofluorescence showing expression of CD31 (endothelium) and α-SMA (pericytes) in DD nodules. Scale bar is 10 μm. (n = 6 DD patients). (D) Confocal images of immunofluorescence showing morphology and distribution of α-SMA^+ pericytes in DD nodules. Scale bar is 10 μm. (n = 6 DD patients). (E) Box and whisker plot showing quantification of vessel density in myofibroblast foci and fibroblast areas in DD nodules. 2-sided unpaired t-test. Scale bar 50 μm (n = 6 DD patients, 3 independent experiments).

A

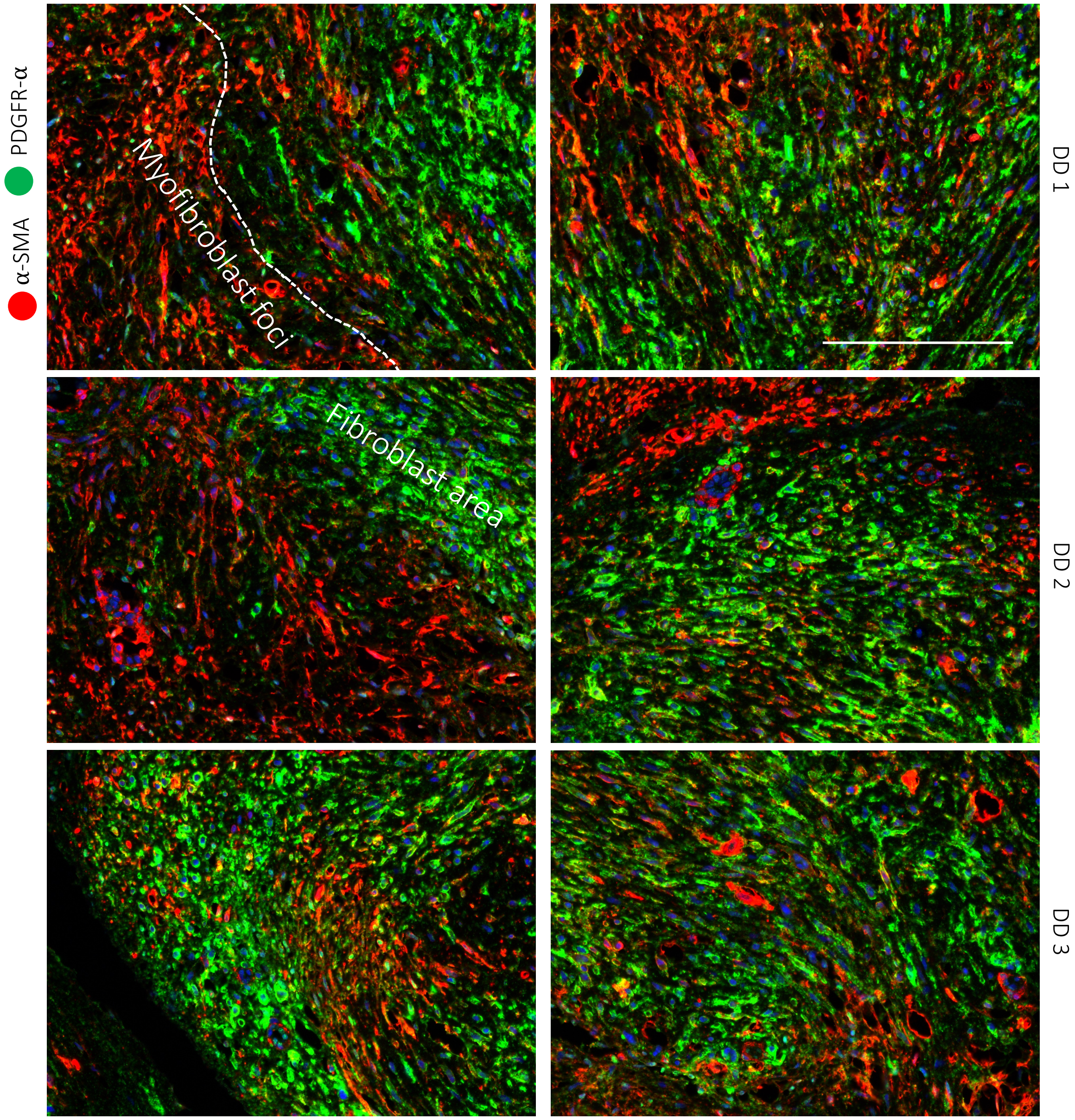
α-SMA
PDGFR-α
Myofibroblast foci
Fibroblast area
DD 1
DD 2
DD 3

Fig. S3. Discrete stromal cell microenvironments in Dupuytren’s nodules.

(A) Confocal images of immunofluorescence in six independent patients showing expression of α-SMA and PDGFR-α in DD nodules. Scale bar is 50 µm.

A

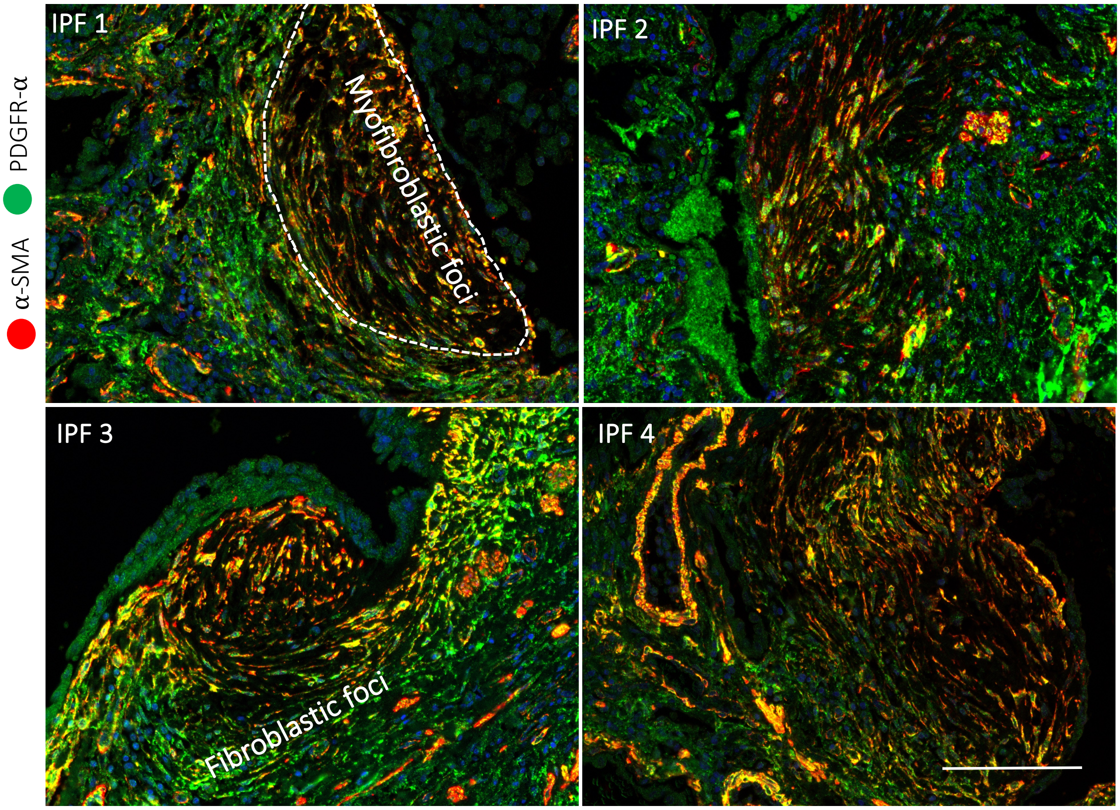

PDGFR-α
α-SMA
IPF 1
Myofibroblastic foci
IPF 2
IPF 3
Fibroblastic foci
IPF 4

Fig S4. Discrete stromal cell microenvironments in IPF

(A) Confocal images of immunofluorescence in four independent patients showing expression of α-SMA and PDGFR-α in human IPF. Scale bar is 70 μm.

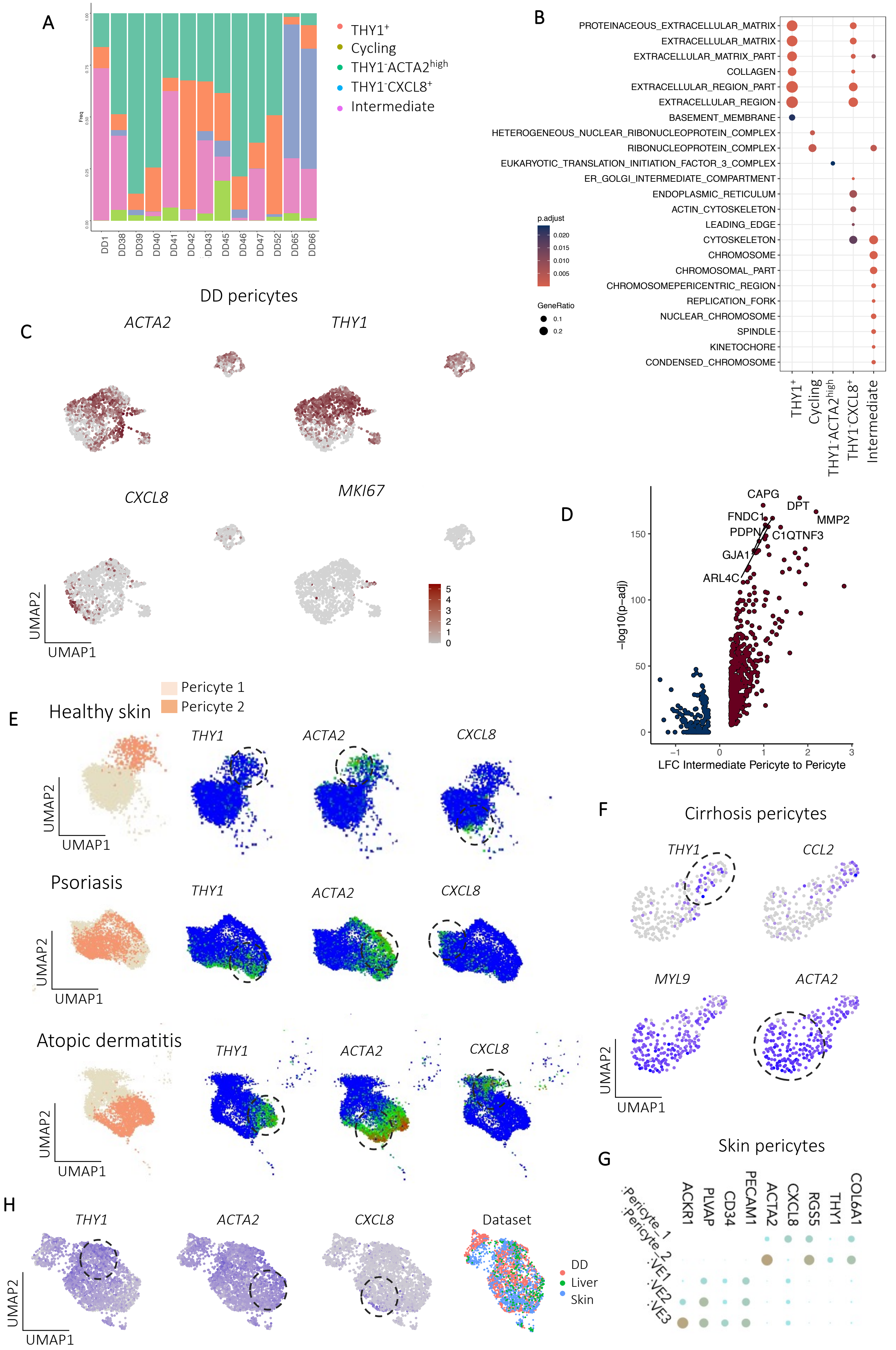

A
THY1+
Cycling
THY1-ACTA2high
THY1-CXCL8+
Intermediate
DD1
DD38
DD39
DD40
DD41
DD42
DD43
DD45
DD46
DD47
DD52
DD65
DD66
B
PROTEINACEOUS_EXTRACELLULAR_MATRIX
EXTRACELLULAR_MATRIX
EXTRACELLULAR_MATRIX_PART
COLLAGEN
EXTRACELLULAR_REGION_PART
EXTRACELLULAR_REGION
BASEMENT_MEMBRANE
HETEROGENEOUS_NUCLEAR_RIBONUCLEOPROTEIN_COMPLEX
RIBONUCLEOPROTEIN_COMPLEX
EUKARYOTIC_TRANSLATION_INITIATION_FACTOR_3_COMPLEX
ER_GOLGI_INTERMEDIATE_COMPARTMENT
ENDOPLASMIC_RETICULUM
ACTIN_CYTOSKELETON
LEADING_EDGE
CYTOSKELETON
CHROMOSOME
CHROMOSOMAL_PART
CHROMOSOMEPERICENTRIC_REGION
REPLICATION_FORK
NUCLEAR_CHROMOSOME
SPINDLE
KINETOCHORE
CONDENSED_CHROMOSOME
p.adjust
0.020
0.015
0.010
0.005
GeneRatio
0.1
0.2
DD pericytes
C
ACTA2
THY1
CXCL8
MKI67
UMAP2
UMAP1
5
4
3
2
1
0
D
CAPG
DPT
FNDC1
MMP2
PDPN
C1QTNF3
GJA1
ARL4C
-log10(p-adj)
LFC Intermediate Pericyte to Pericyte
E
Healthy skin
Pericyte 1
Pericyte 2
Psoriasis
Atopic dermatitis
F
Cirrhosis pericytes
CCL2
MYL9
G
Skin pericytes
ACKR1
PLVAP
CD34
PECAM1
RGS5
COL6A1
Pericyte_1
Pericyte_2
VE1
VE2
VE3
H
Dataset
DD
Liver
Skin

Fig. S5. Five subsets of pro-fibrotic pericytes housing an intermediate population

(A) Bar plot of single cell RNA-seq showing the proportions of pericyte subsets in DD patients as a total of all pericytes. (*n* = 12 DD patients). (B) Dot plot showing pathways enriched in pericytes in single cell RNA-seq. Gene ratio is the number of genes found in pathways and p.adjust is adjusted p-value (2-sided Wilcoxon Rank Sum test, BH FDR-correction). (C) UMAP projections of single cell RNA-seq of DD pericytes coloured by z-score expression of marker genes of major subsets (*ACTA2*, *THY1, CXCL8* & *MKI67*). (*n* = 12 DD patients). (D) Volcano plot showing differentially expressed genes (2-sided Wilcoxon Rank Sum Test) between pericytes and intermediate pericytes in single cell RNA-seq with FDR corrected adjusted *p*-values (BH correction). (E) UMAP projections of single cell RNA-seq of skin pericytes coloured clusters and by z-score expression of marker genes of major subsets (*ACTA2*, *THY1* & *CXCL8*). (F) UMAP projections of single cell RNA-seq of liver pericytes coloured by z-score expression of marker genes of major subsets (*ACTA2* & *THY1*). (G) Dot plot of skin pericytes scRNA-seq showing expression of general pericyte markers (*COL6A1* & *RSG5*) and those of specific subsets (*THY1*, *ACTA2* & *CXCL8*). (H) UMAP projections of cross tissue pericyte single cell RNA-seq atlas of skin, liver and hand pericytes coloured clusters and by z-score expression of marker genes of major subsets (*ACTA2*, *THY1* & *CXCL8*).

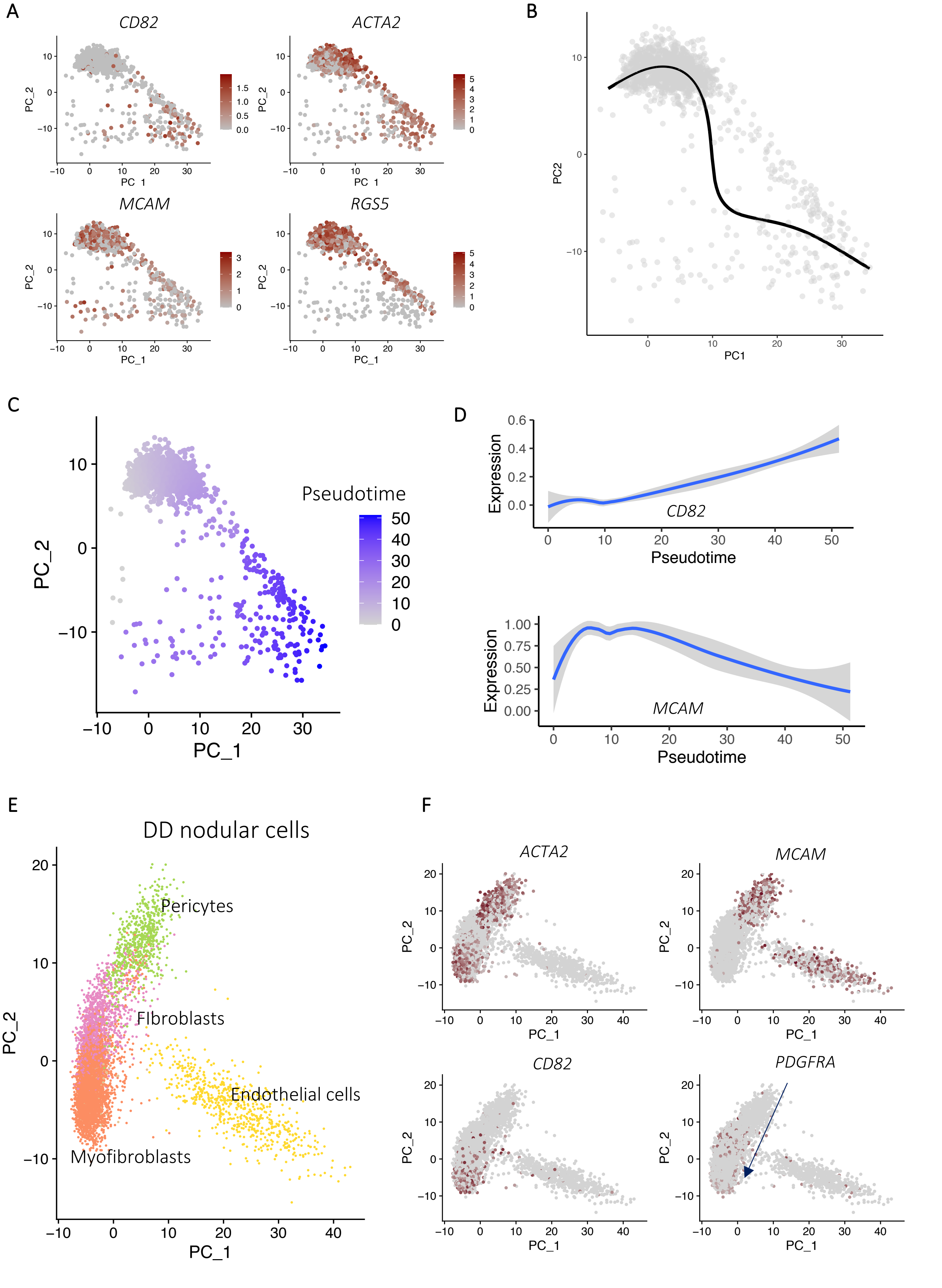

A
CD82
ACTA2
MCAM
RGS5
PC_1
PC_2
B
PC1
PC2
C
Pseudotime
PC_1
PC_2
D
CD82
MCAM
Expression
Pseudotime
E
DD nodular cells
Pericytes
Fibroblasts
Endothelial cells
Myofibroblasts
PC_1
PC_2
F
ACTA2
MCAM
CD82
PDGFRA
PC_1
PC_2

Fig. S6. Pseudotemporal ordering of pro-fibrotic pericytes

(A) Principal component analysis (PCA) of pericyte single cell RNA-seq showing the expression of selected genes in log(UMI+1) (*n*=12 DD patients). PCA is a linear dimensional reduction method and the first two principles components capture a putative differentiation pathway towards the intermediate subset. (B) PCA embedding of pericyte single cell RNA-seq showing overlay of principle curve fit. Principle curve used to assign cells along pseudotime order. (C) PCA embedding of pericyte single cell RNA-seq coloured by pseudotime. Pseudotime is defined by the principal curve fit to PCA coordinates (smoother = 'lowess', *f* = 1/3). (D) Line plots showing the expression (*loess* smoothed normalized expression +|- SE) of selected genes along modelled pseudotime (slingshot pseudotime) in single cell RNA-seq of pericytes (*n* = 12 DD patients). Pseudotime was defined by fitting a principal curve along the first two diffusion map coordinates. (E) PCA embedding of entire DD dataset single cell RNA-seq coloured by major cell types (*n*=6, *k*= 7,234 cells). (F) PCA embedding of entire DD dataset single cell RNA-seq coloured by the expression of lineage markers in log(UMI+1) (*n*=12 DD patients). *ACTA2* marks pericytes and myofibroblasts. *MCAM* marks endothelial cells and pericytes. CD82 marks myofibroblasts and *PDGFRA* marks fibroblasts. Arrows represents the trajectory moving from pericytes to myofibroblasts. The PCA plot demonstrates a trajectory along PC2 that captures a pericyte to fibroblasts to myofibroblast transition marked by the arrow.

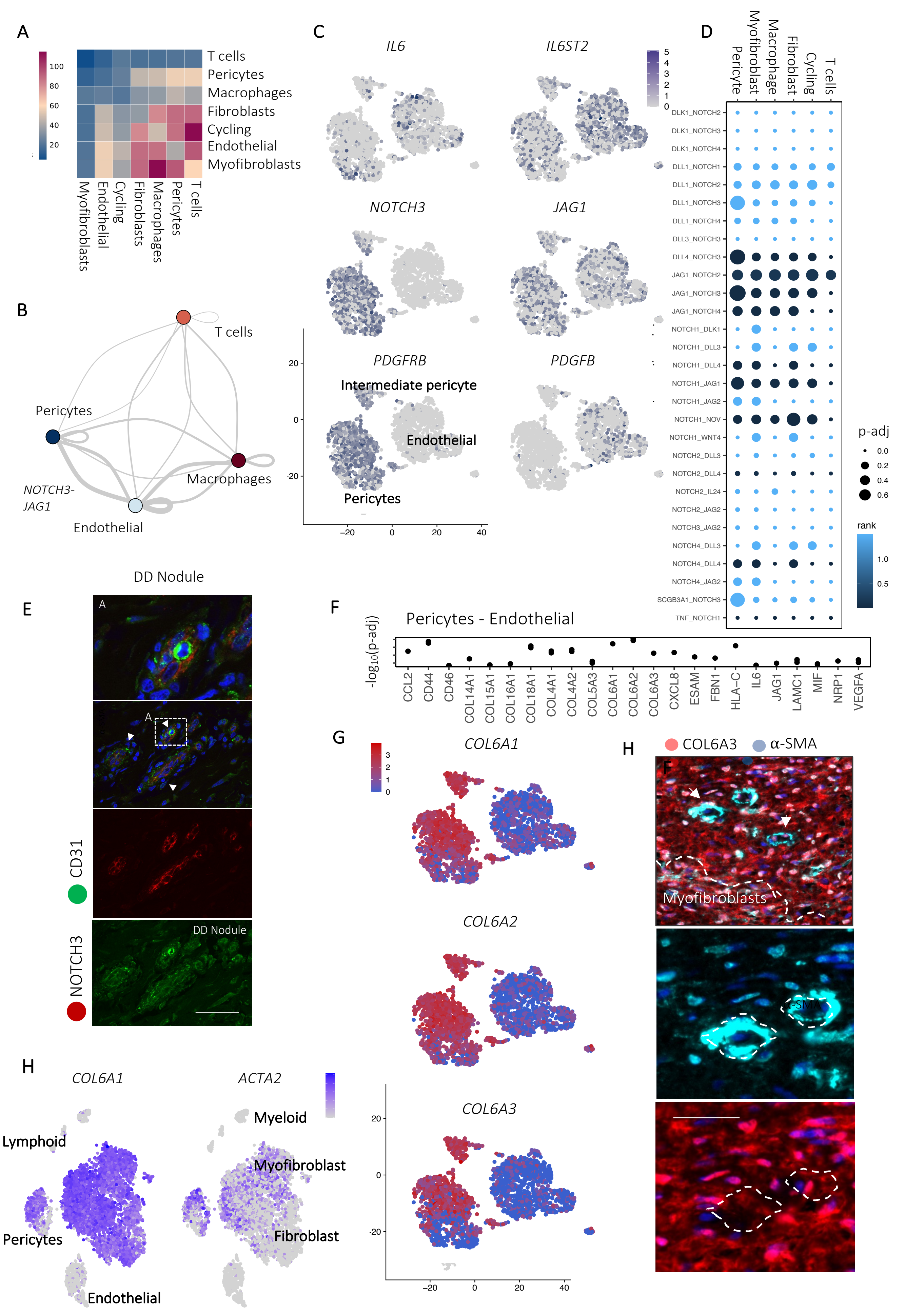

A
T cells
Pericytes
Macrophages
Fibroblasts
Cycling
Endothelial
Myofibroblasts
B
T cells
Pericytes
Macrophages
NOTCH3-JAG1
Endothelial
C
IL6
IL6ST2
NOTCH3
JAG1
PDGFRB
PDGFB
Intermediate pericyte
Endothelial
Pericytes
D
Pericyte
Myofibroblast
Macrophage
Fibroblast
Cycling
T cells
DLK1_NOTCH2
DLK1_NOTCH3
DLK1_NOTCH4
DLL1_NOTCH1
DLL1_NOTCH2
DLL1_NOTCH3
DLL1_NOTCH4
DLL3_NOTCH3
DLL4_NOTCH3
JAG1_NOTCH2
JAG1_NOTCH3
JAG1_NOTCH4
NOTCH1_DLK1
NOTCH1_DLL3
NOTCH1_DLL4
NOTCH1_JAG1
NOTCH1_JAG2
NOTCH1_NOV
NOTCH1_WNT4
NOTCH2_DLL3
NOTCH2_DLL4
NOTCH2_IL24
NOTCH2_JAG2
NOTCH3_JAG2
NOTCH4_DLL3
NOTCH4_DLL4
NOTCH4_JAG2
SCGB3A1_NOTCH3
TNF_NOTCH1
p-adj
rank
DD Nodule
E
CD31
NOTCH3
F
Pericytes - Endothelial
-log10(p-adj)
CCL2
CD44
CD46
COL14A1
COL15A1
COL16A1
COL18A1
COL4A1
COL4A2
COL5A3
COL6A1
COL6A2
COL6A3
CXCL8
ESAM
FBN1
HLA-C
IL6
JAG1
LAMC1
MIF
NRP1
VEGFA
G
COL6A1
COL6A2
COL6A3
H
COL6A3
α-SMA
Myofibroblasts
H
COL6A1
ACTA2
Lymphoid
Pericytes
Endothelial
Myeloid
Myofibroblast
Fibroblast

Fig. S7. Coupling of the fibrotic vasculature

(A) Heatmap of cell-cell interaction analysis in single cell RNA-seq showing the number of significant (p-adjust < 0.05) interactions between cell types in DD nodules (*n* = 12 DD patients). (B) Network plot showing interactions between cell types. Line width is weighted by the number of significant interactions. (C) tSNE projections of single cell RNA-seq showing RNA expression (log(UMI+1)) in pericytes and endothelial cells of selected genes in significantly enriched pathways between cell types. (D) Dot plot showing RNA expression of receptor-ligand pairs in significantly enriched pathways following interaction analysis of single cell RNA-seq. Rank is the receptor-ligand pair rank in pathways and p.adjust is adjusted p-value (2-sided Wilcoxon Rank Sum test, BH FDR-correction). (E) Confocal images of immunofluorescence showing NOTCH3 expression in pericytes lining the endothelium (CD31) in DD nodules (*n* = 3 DD patients). Scale bar 30 µm. (F) Dot plot showing pericyte genes mediating the significant pericyte-endothelial cell interactions. p.adjust is adjusted p-value (2-sided Wilcoxon Rank Sum test, BH FDR-correction). (G) tSNE projections of single cell RNA-seq showing RNA expression (log(UMI+1)) in pericytes and endothelial cells of collagen 6 transcripts. (H) Confocal images of immunofluorescence showing COL6A3 and α-SMA expression in pericytes in DD nodules (*n* = 3 DD patients). Scale bar 10 µm. (H) tSNE projections of single cell RNA-seq showing RNA expression (log(UMI+1)) in DD nodular cells of *ACTA2* and *COL6A1*.

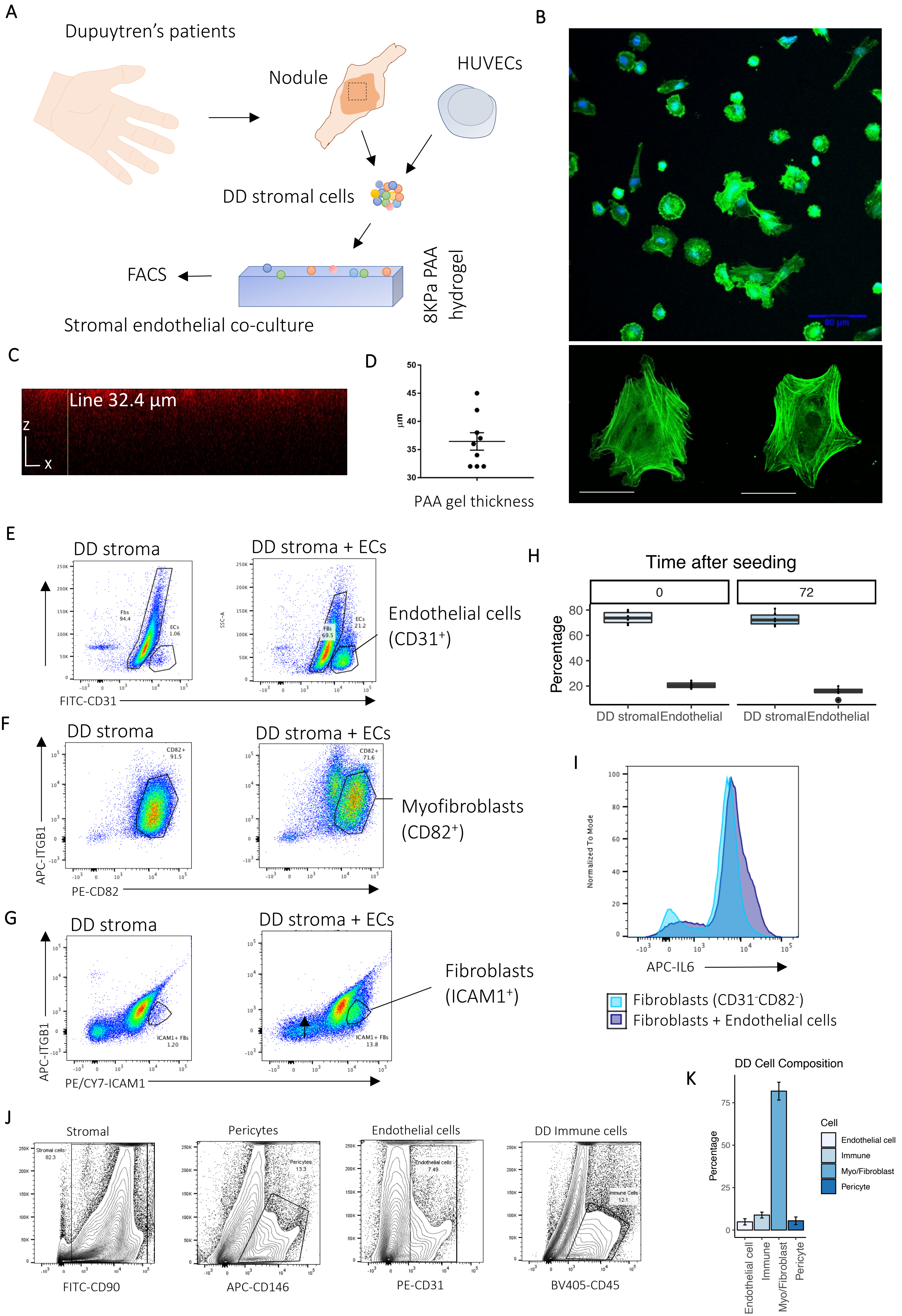
A
Dupuytren's patients
Nodule
HUVECs
DD stromal cells
FACS
8KPa PAA hydrogel
Stromal endothelial co-culture
B
C
Line 32.4 µm
Z
X
D
µm
PAA gel thickness
E
DD stroma
DD stroma + ECs
Fbs 94.4
ECs 1.06
FBs 69.5
ECs 21.2
SSC-A
Endothelial cells (CD31+)
FITC-CD31
F
DD stroma
DD stroma + ECs
CD82+ 91.5
CD82+ 71.6
Myofibroblasts (CD82+)
APC-ITGB1
PE-CD82
G
DD stroma
DD stroma + ECs
ICAM1+ FBs 1.20
ICAM1+ FBs 13.8
Fibroblasts (ICAM1+)
APC-ITGB1
PE/CY7-ICAM1
H
Time after seeding
0
72
Percentage
DD stromal
Endothelial
I
Normalized To Mode
APC-IL6
Fibroblasts (CD31-CD82-)
Fibroblasts + Endothelial cells
J
Stromal
Stromal cells 82.3
Pericytes
Pericytes 13.3
Endothelial cells
Endothelial cells 7.49
DD Immune cells
Immune Cells 12.1
FITC-CD90
APC-CD146
PE-CD31
BV405-CD45
K
DD Cell Composition
Percentage
Cell
Endothelial cell
Immune
Myo/Fibroblast
Pericyte

Fig. S8. Co-culture system with DD stromal cells and endothelial cells.

(A)Schematic illustrating experimental protocol for single cell profiling of Dupuytren's nodule. (B) Confocal image of Phalloidin-488 staining of DD stromal cells 72hrs after seeding on 8KPa polyacrylamide (PAA) hydrogel showing cell attachment, spreading and stress fibre formation. (C) Image of z-stack acquisition of PAA hydrogel with fiducial marker beads (FluoSpheres MicroSpheres 0.2 μm) showing thickness of PAA hydrogels (D) Scatter plot showing PAA gel thickness (mean = 36.2 μm, *n* = 9 intendent experiments). (E-G) Density plots of flow cytometry analysis for co-culture of freshly isolated (E) DD nodular cells and endothelial cells showing proportion of (F) myofibroblasts ($CD82^{+}ITG\text{-}\beta1^{high}$) and (G) fibroblasts ($ICAM1^{+}ITG\text{-}\beta1^{high}$) (*n* = 8 DD patients). (H) Boxplot showing flow cytometry analysis of the percentage of endothelial cells and DD stromal cells ($CD45^{-}$) before (0hrs) and after (72hrs) co-culture assay (*n*=8 DD patients). (I) Histogram showing flow cytometry analysis of IL6 expression in fibroblasts ($ICAM1^{+}ITG\text{-}\beta1^{low}$) with and without endothelial cells (*n* = 6 DD patients). (J) Density plots of flow cytometry analysis of isolated DD nodular cells showing proportion of stromal ($CD45^{-}THY1^{+}$), pericytes ($CD45^{-}CD31^{-}CD146^{+}$), endothelial cells ($CD45^{-}CD31^{+}CD146^{-}$) and immune cells ($CD45^{-}$). (K) Bar plot of flow cytometry analysis showing the proportion of cell types in freshly isolated DD nodular cells (*n*=8 DD patients).
